# Autism trait prevalence in treatment seeking adolescents and adults attending specialist gender services

**DOI:** 10.1192/j.eurpsy.2020.23

**Published:** 2020-03-02

**Authors:** Katrin Lehmann, Michael Rosato, Hugh McKenna, Gerard Leavey

**Affiliations:** 1 Bamford Centre for Mental Health & Wellbeing, Ulster University, Northern Ireland, United Kingdom; 2 Institute of Nursing and Health Research, Ulster University, Jordanstown, Northern Ireland, United Kingdom

**Keywords:** Autism, gender dysphoria, prevalence, transgender

## Abstract

**Background.:**

To assess the prevalence of autism traits in individuals accessing gender affirming treatments, we conducted a cross-sectional survey in the regional specialist gender services in Northern Ireland.

**Methods.:**

One hundred and twenty-three individuals (38 adolescents and 69 adults) currently attending or who previously attended specialist gender services in Northern Ireland were recruited. Fifty-six individuals assigned male at birth (AMAB) and 66 individuals assigned female at birth (AFAB) took part in the study. Main outcome measures: Autism Quotient (AQ), Cambridge Behavior Scale (EQ), and RAADS-14.

**Results.:**

Autism trait prevalence rates of 19.5% (AQ); 25.4% (RAADS-14); and 35.8% (poor empathy traits). A combined measure comprising all three provided a prevalence of 17.2%. There were no mean differences in the scores between AMAB (assigned male at birth) individuals and AFAB (assigned female at birth) individuals.

**Conclusions.:**

Autism traits present additional challenges during the assessment and treatment of individuals with gender dysphoria. Autism screening tools can aid in the identification of individual with additional needs.

## Introduction

Gender dysphoria refers to the distress experienced due to the incongruence between a person’s inner perception of their gender and an incongruous bodily reality. Symptoms of gender dysphoria include strong and persisting cross-gender identification and a desire to be of the other gender [1]. This can include distress associated with specific body parts of the birth gender and distress associated with having to dress or act in ways associated with the birth-assigned gender role. The experience of gender-related distress is unique to each individual and, based on clinical observations, can manifest in very different forms. The treatment of gender related distress is based on World Professional Organization for Transgender Health (WPATH) guidance [2]. Currently, after careful psychological assessment, medical interventions for adolescents and adults are available. Some individuals with gender incongruence may not experience distress or have a wish to live as the other gender [3]. How gender dysphoria is conceptualized has changed dramatically over time and with controversy [4]. To this day, little evidence supports biological or psychological theories on gender dysphoria with societal and political debates continuing to be highly polarized [5].

Like gender dysphoria, autism has been understood within a medical model—a disorder within the DSM and ICD classification system [6,7]. Here autism, incorporating autistic spectrum disorders and Asperger’s syndrome (AS), is characterized by qualitative impairment in social interaction and communication skills, as well as stereotypic behaviors and limitations on activities and interests [8]. Autism traits can be assessed using a number of screening tools, which can identify those with higher likelihood of autism features. Within the literature some have questioned whether the neurological differences in autism or AS have been socially constructed and presented as a disorder [9]. Autism and AS have been dominated by theories of mind deficit and pathological deviance from expected developmental norms [10]. Using a medical approach to a developmental issue has been criticized on the basis that others tend to view the individual through a diagnostic lens, thereby limiting expectations of the diagnosed individual as a result [9]. Theory of mind or the ability to empathize with others is often seen as lacking in individuals with autism or AS. Milton [10] suggests that encounters of empathy are often perceived as threatening for the autistic person and that empathy is bi-directional—located both in the autistic and nonautistic person as a double empathy problem [11].

Referrals to specialist gender services in Great Britain have markedly increased over recent years [12]. Moreover, the prevalence of gender dysphoria and autism has also increased [13,14], possibly explained by improved access to diagnostic services and increased awareness of autism features among clinicians and the general public. Likewise, increased prevalence of gender dysphoria could be related to higher visibility and tolerance toward transgender individuals in conjunction with a widening of diagnostic criteria [15].

The co-occurrence of autism and gender dysphoria presents significant diagnostic and treatment challenges given the associated social, adaptive, self-awareness, communication, and executive function complexities [16]. Misrecognition of autism traits can lead to individuals being undertreated, misunderstood, and unsupported [17]. The double empathy problem [10] may create additional challenges in the delivery of services as most clinicians working in specialist gender services are neurotypical.

### Aim

To examine prevalence rates of co-occurring autism traits and gender dysphoria in a clinical sample. Explore if there are differences between AMAB and AFAB individuals.

## Background

### Children and adolescents

de Vries et al. [18] conducted a cohort study in the Netherlands, with 204 adolescents, which used DISCO, a validated diagnostic assessment tool completed with parents, as part of a multidisciplinary diagnostic assessment to establish an autism diagnosis. This reported an autism prevalence of 7.8% among young people with gender dysphoria seeking gender affirming treatments, nearly 10 times that of the general population. There was no control group in the study. This study provided a robust assessment of autism, but the use of diagnostic assessment interviews by trained clinical diagnosticians required significant time and service resources. Di Ceglie et al. [19] conducted a case–control study using the parent completed empathizing and systemizing quotients (EQ and SQ) [20] in a sample of 25 adolescents with gender dysphoria and 156 adolescent controls from the general population. The study reported lower empathy levels among females assigned at birth and suggested that those experiencing gender dysphoria might benefit from additional social skills training to develop empathy [19]. While this study was unable to report autism trait prevalence, the exploration of empathy levels in autism added to the understanding of needs in individuals presenting to clinical services.

Both Skagerberg et al. [21] and VanderLaan et al. [22], conducted studies using the parent completed social responsiveness scale (SRS): the former, a case–control study with 166 parents of children and adolescents referred due to gender dysphoria—reported no significant differences between mean SRS scores of cases and controls; and the latter, a cohort study with 49 parents of gender referred children who completed the SRS, did not report autism trait prevalence directly, but suggested that increased birth-weight was predictive of increased autism traits, and that increased autism traits were predictive of increased gender non-conformity in children. Higher levels of gender variance have also been reported in the autism population. Strang et al. [23] found that children and young people with autism were 7.6 times more likely to have gender variance compared to non-referred controls. George and Stokes [24] also found significantly higher rates of gender dysphoria in their sample of 309 autistic adults compared to their control sample of 261 neurotypical adults.

Kaltiala-Heino et al. [25] conducted a cohort study using chart review data of 47 adolescents attending a specialist gender clinic in Finland, and reported that 26% had a previous diagnosis of autism recorded in their clinical chart. Van der Miesen et al. [26] conducted a study with parents of 490 children with gender dysphoria, 2,507 parents of neurotypical children and parents of 196 children with Autism using the Children’s Social Behavior Questionnaire (CSBQ), reporting elevated levels of autism traits in children with gender dysphoria [26].

## Adults

Jones et al. [27], using the self-report Autism Quotient (AQ) [28], conducted a case–control study using an online survey of 194 adults transgender individuals recruited from an NHS Gender Identity Clinic website. The comparison groups in this study consisted of 174 individuals from the general population and 125 individuals with a known autism diagnosis. Prevalence of autism symptoms were 1.5% for AMAB individuals and 13.8% for AFAB individuals, using the narrow autism phenotype (NAP) range, which was indicated by AQ scores of 35 and above [27]. Using the more stringent criteria of NAP, compared to scores of 32 and above, used in other studies highlighted that AFAB individuals had an 11-fold increase in the rate of autism traits. Pasterski et al. [29] also used the self-report AQ in a clinical sample of 91 adults and a control group of 840 university students, and reported that 5.5% of their clinical sample scored in the range suggestive of an autism diagnosis.

Heylens et al. [30] conducted a cross-sectional study in a sample of 63 treatment seeking adults using the Social Responsiveness Scale Adults (SRS-A), the AQ, and clinical chart data of 532 adults referred to a specialist gender service over a 5-year period: based on the SRS-A, 27.1% of gender-referred individuals scored within the range of mild/moderate/severe difficulties in relation to social responsiveness and 4.8% scored within a range suggestive of an autism diagnosis [30]. The clinical chart data highlighted that 6.0% of individuals attending the service over a period of 5 years had a certain autism diagnosis [30]. In another study, Vermaat et al. [31] reported mean AQ score in individuals seeking gender affirming treatments which were similar to neurotypical samples, suggesting that the co-occurrence of autism and gender dysphoria may not be as prevalent as suggested.

Nobili et al. [32] conducted a case–control study of 656 treatment-seeking transgender adults and matched cisgender community controls using the AQ-Short, and reported a 36.3% prevalence of autism traits in treatment-seeking adults and 33.2% in cisgender community controls, with no significant difference between the groups. Studies by Jones et al. [27] and Nobili et al. [32] reported higher rates of autism traits in AFAB individuals compared to any other group and suggest that differences between transgender and cisgender individuals are due to high levels of autism traits in AFAB individuals.

### Potential hypotheses on relationship between gender dysphoria and autism

There may be a familial or genetic association between gender dysphoria and autism [33], but the evidence is weak. The *extreme male brain theory* suggests that individuals with autism have patterns of behavior and empathy more closely resembling male patterns [34]: suggested also by lower empathy and higher autistic traits patterns in AFAB individuals [19]. Rigidity related to an autism diagnosis suggests a potential explanatory factor, with individuals not reaching levels of flexibility required to deal with gender variant feelings [18]. Obsessionality in gender dysphoria and autism have also been noted. Other explanations include association between social impairment, stress, and sexual minority status [18]; developmental problems related to childhood maltreatment and trauma and body image [35]. Additionally, Walsh et al. [36] suggest that the relationship between autistic resistance to social conditioning, rejection of cisgenderist (opposite to transgenderist) norms in combination with below-typical concern of social norms could explain elevated rates of trans identity.

## Methodology

### Procedure

With a population of 1.81 million usual residents [37], Northern Ireland has two dedicated regional specialist gender services, one for children and young people and another service for adults based in Belfast. Young people who reach the age of 18 years are transferred from the adolescent to the adult specialist gender service. Individuals who move away from Northern Ireland are referred to their preferred local service in Great Britain. Individuals in Northern Ireland can access hormonal interventions locally but access to gender affirming surgical interventions is arranged on a case by case basis outside Northern Ireland.

Potential participants aged 16 years and over were initially approached by the clinical teams from the regional adult and young person specialist gender services in Northern Ireland. Detailed written information about the cross-sectional survey was provided at this stage. Interested participants provided written consent to be contacted directly by the researcher. A time and place were arranged to complete the survey. Participants provided written informed consent to take part in the study.

Severe cognitive impairment or those experiencing acute psychotic symptoms were excluded.

### Recruitment

Participants were recruited through specialist services, and trans- and LGBT-specific organizations. Experts-by-experience provided signposting within the wider community. Posters and leaflets for the study were displayed in the clinical service waiting rooms as well as community spaces and the meeting rooms of trans and LGBT organizations. While the majority of individuals approached by clinicians (*N* = 140) agreed to participate in the study, 10% declined to take part.

### Ethical approvals

This analysis is part of a wider mixed methods project—gender identity-finding and transforming services (GIFTS) study. Ethical approval for the project was gained through (a) ORECNI on June 15, 2017 (REC reference: 17/NI/0069 Protocol number: 17/0017 IRAS project ID: 220537) and (b) trust governance approval was given on July 6, 2017 (HSC Trust reference: 16204GL-SP).

### Data collection instruments

Experts-by-experience assisted the planning and delivery of the study including questionnaire design. All had experience of gender dysphoria and two also had lived experience of autism.

## Measures

### Sociodemographic

Age, postcode, sex assigned at birth, self-description, ethnic or cultural background, highest education/training levels, religious denomination, significance of faith, parental occupation/participant occupation, sexual orientation, sexual attraction, and awareness of gender incongruence. Sexual orientation was based on free text information provided by participants and grouped into four categories based on another study [38].

### Service-related

First contact with specialist gender services and transition (social transition, endocrine treatments, and gender affirming surgical procedures). Table 1 provides an overview of the outcome and potential explanatory variables in this study. Treatment status was not controlled for in this study and participants were at various stages of gender affirming interventions.

**Table 1.** Sociodemographic, sexual orientation, and clinical intervention characteristics of study participants.

		All *N* (%)
Current age (total = 123)	Adolescents < 18	38 (30.9)
	Adults > 18	85 (69.1)
Sex at birth (total = 123)	Male	57 (46.3)
	Female	66 (53.7)
Self-identified gender (total = 123)	Male	42 (34.2)
	Female	33 (26.8)
	Transmale/transfemale	42 (34.2)
	Non-binary	6 (4.9)
Respondent education (total = 118)	Degree or higher	6 (5.1)
	Intermediate	83 (70.3)
	No qualifications	29 (24.6)
Respondent occupational social class (total = 118)	Professional	33 (28.0)
	Intermediate	19 (16.1)
	(Semi-)routine	35 (29.7)
	Other	31 (26.3)
Occupational social class of family (total = 121)	Professional	53 (43.8)
	Intermediate	23 (19.0)
	(Semi-)routine	39 (32.2)
	Other	6 (5.0)
Sexual orientation (total = 121)	Monosexual	48 (39.7)
	Plurisexual	58 (47.9)
	Asexual	7 (5.8)
	Other/unsure	8 (6.6)
Cross-sex hormones (total = 122)	Yes	48 (39.3)
	No	74 (60.7)
Gender affirming surgery (total = 121)	Yes	95 (78.5)
	No	26 (21.5)
Desire to access cross-sex hormones (total = 122)	Yes	118 (96.7)
	No	4 (3.3)
Desire to have gender affirming surgery (total = 122)	Yes	117 (95.9%)
	No	5 (4.1%)

### Autism screening tools

#### ***Autism Quotient*** [28]

The AQ was developed as a brief self-administered questionnaire to assess the degree of autistic traits in an individual [28]. The AQ is used for adolescents (>15.5 years) and adults, with (AQ-adolescent) for children and adolescents (9.8–15.4 years) [39]. The AQ was developed as a screening tool to identify those who might benefit from a clinical diagnostic assessment. The AQ contains 50 questions covering the following domains: social skill, attention switching, attention to detail, communication, and imagination [28]. Items are rated on a 4-point Likert scale between “definitely agree” to “definitely disagree.” Items on each domain are given the score of 1 if the individual identifies the autistic like behavior on the item either as mildly or strongly [28]. To avoid response bias approximately 25 items were worded to produce an agree response with remaining items producing a disagree response. After this process, questions were mixed up related to responses and domains [28]. Scores are totaled at the end of the questionnaire to indicate the degree of autistic traits with the highest possible score of 50. A score of 32 and higher indicates a higher likelihood of clinically significant autistic traits which may require further clinical diagnostic assessments. The AQ demonstrated reasonable construct validity and showed moderate to high alpha coefficients [28] in the original study. Further studies [40] reported satisfactory to near-satisfactory reliabilities of the AQ in U.K. samples, with lower reliabilities demonstrated in U.S. samples [41]. Both contained cisgender rather than transgender samples.

#### ***Cambridge Behavior Scale*** [42]

The EQ [42] was developed as a short, self-administered, and easy to score instrument to measure how individuals can understand the intentions of others and predict how others might think or react in social situations. Autism has been described as an empathy disorder by some [43] based on mindreading difficulties for individuals. The EQ contains 60 questions related to cognitive and affective empathy [44] and empathic behaviors, with 40 questions directly related to measuring empathy, and 20 unrelated filler questions. Cognitive empathy is related to perspective taking and understanding of why others behave in a certain way, whereas affective empathy is based on the emotions a person feels by observing other people [45]. Responses are scored one for mildly empathic and two for strongly empathic. A score of below 32 is likely to indicate lower levels of empathy which can be associated with autism. Questions were worded to produce a mixture of disagree and agree responses to avoid response bias [42]. The EQ has been evidenced to have high retest-reliability (*r* = 0.97) and has been validated as an instrument in adults with AS [42].

#### ***RAADS-14*** [46]

The RAADS-14 [46] was developed as a screening tool to identify individuals already attending mental health services who may require a diagnostic assessment for autism. While the authors acknowledge the usefulness of the AQ [28] and RAADS-R [47], both instruments were perceived to be too lengthy for screening purposes in the clinical setting [46]. The RAADS-14 reduces the number of questions from the RAADS-R from 80 to 14 items. The RAADS-14 items are in the domains of mentalizing deficits, social anxiety, and sensory reactivity [46]. Items are scored based on the same 4-point Likert scale used for the RAADS-R. The RAADS-14 takes only minutes to complete and demonstrated internal consistency above 0.7 and adequate construct and convergent validity across all domains [46]. Participants in the GIFTS study were able to complete the RAADS-14 without difficulties and the change from the RAADS-R reduced the overall time required to participate in the study significantly. A score of 32 or above on the RAADS-14 is seen as suggestive of possible autism/autism traits.

Autism screening tools rather than diagnostic tools may lead to false positives [48]. To reduce the possibility of false positives a more conservative indicator of autism was used based on a combination of all three: those scoring 32 or above on both the AQ [28] and RAADS-14 [46] measure and below 32 on the EQ [42] were classed as exhibiting autism traits.

### Statistical analysis

All data were analyzed using STATA 14.2. All raw data from the questionnaires were inputted manually into STATA and checked. *T*-tests were used for continuous data, to test hypotheses related to differences in continuous variables. Mean scores for the AQ, Cambridge Behavior Scale (EQ), and RAADS-14 were evaluated using independent sample *t*-tests. The significance level was set at *p* = <0.05. Chi-square tests were used for discrete data to assess if the proportions of individuals in the Autism traits group differed from other participants.

## Results

### Participant demographics

Table 1 outlines the characteristics of study participants. Overall, the ratio of adults to adolescents in the study was 2:1. The proportion of AFAB individuals was slightly higher (53.7%) compared to AMAB individuals (46.3%). Using an independent samples *t*-test (*t* = 4.41; *df* = 121), there were statistically significant differences (*p* < 0.00) in mean ages between AMAB individuals (mean age: 33.8 years; standard deviation [SD]: 15.7; 95% confidence intervals [CI]: 29.6–38.0) and AFAB individuals (mean age: 23.2 years; SD: 10.8; 95% CI: 20.5–25.8).

Participants self-identified as male (34.2%), female (26.8%), transmale/transfemale (34.2%), or non-binary (4.9%). Analysis of variance (ANOVA) between groups (*f* = 5.3; *df* = 3) and within groups (*f* = 5.3; *df* = 119) highlighted statistically significant (*p* = 0.002) differences in mean ages, with self-identified males (mean age: 23.6 years; SD: 12.0), self-identified females (mean age: 35.7 years; SD: 16.9), transmales/transfemales (mean age: 26.3 years; SD: 11.9), and non-binary individuals (mean age: 31; SD: 14.2).

Because of missing information, educational attainment is based on 118 participants: most reported achieving intermediate qualifications (70.3%), with a small number reporting degree or higher (5.1%), and a quarter (24.6%) reporting having no formal qualifications. Respondent occupational social class was reported as professional (28%), intermediate (16.1%), (semi)-routine (29.7%), and other (26.3%). Respondent family occupational social class was reported as professional (43.8%), intermediate (19.0%), (semi)-routine (32.2%), and other (5.0%).

Participants self-classified as: monosexual (39.7%), plurisexual (47.9%), asexual (5.8%), or other/unsure (6.6%). ANOVA between groups (*f* = 1.6; *df* = 3) and within groups (*f* = 1.6; *df* = 110) highlighted no statistically significant difference (*p* = 0.2) in mean age of participants identifying as monosexual (mean age: 30.0 years; SD 15.6), plurisexual (mean age: 24.4 years; SD: 11.4), asexual (29.4 years; SD: 14.0), and unsure/questioning (25.3 years; SD: 7.9).

One hundred and eighteen participants wanted to access cross-sex hormones (96.7%) and 117 participants (95.9%) wished to access gender affirming surgery. None of the adolescents (*n* = 37) had commenced cross-sex hormones, while 48 out of 85 (56.5%) adults had commenced gender affirming hormonal treatment. Adult participants, 26 out of 85 overall (30.6%, 26/85), had had at least one gender affirming surgical procedure, while 59 adult participants (69.4%, 59/85) had no surgical treatment to date. Five adult participants (5.9%, 5/85) did not wish to access any gender affirming surgical interventions.

Based on independent samples *t*-test (*t* = −4.24; *df* = 120), there were statistically significant differences (*p* < 0.00) in mean ages between individuals who had not accessed cross-sex hormones (mean age: 24.3 years; SD: 12.6; 95% CI: 21.3–27.2) and individuals who had accessed cross-sex hormones (mean age: 34.3 years; SD: 14.7; 95% CI: 30.0–38.6). Based on independent samples *t*-tests, there were also statistically significant differences (*p* < 0.00) in mean ages between individuals who had not accessed gender affirming surgical interventions (mean age: 25.5 years; SD: 13.3; 95% CI: 22.8–28.3) and individuals who had accessed gender affirming surgical interventions (mean age: 38.4 years; SD: 13.4; 95% CI: 33.0–43.8).

### Prevalence of autism traits

Prevalence of autism traits based on the AQ was 19.5%. Mean AQ scores overall were 23.00 (SD: 9.77; range: 0–45). Using independent *t*-tests we found no significant difference (*df* = 121; *p* = 0.50) in mean AQ scores between AMAB individuals (mean: 22.4; SD: 9.6; 95% CI: 19.8–24.9) and AFAB individuals (mean: 23.5; SD: 9.9; 95% CI: 21.1–26.0). The Cambridge Behavior Scale (EQ) showed a low empathy prevalence of 35.8% in the sample. Mean scores overall were 38.22 (SD: 14.4; range: 0–67). Based on independent *t*-tests, there were no significant differences (*df* = 121; *p* = 0.37) in mean scores between AMAB individuals (mean: 37.0; SD: 15.4; 95% CI: 32.9–41.0) and AFAB individuals (mean: 39.3; SD: 13.4; 95% CI: 36.0–42.6). Using the RAADS-14, we detected an autism trait prevalence of 25.4% in this study. Overall mean scores in the RAADS-14 were 21.9 (SD: 12.8; range: 0–55) 95% CI: 19.57–24.16). Based on independent *t*-tests there were no significant mean differences (*df* = 120; *p* = 0.58) between AMAB individuals (mean: 21.2; SD: 12.6; 95% CI: 17.8–24.5) and AFAB individuals (mean: 22.5; SD: 13.1; 95% CI: 19.2–25.7). The combined score gave an autism trait prevalence of 17.2% in this sample.

### Autism trait prevalence-comparison based on demographic factors

Table 2 using Pearson’s chi-square tests (*df* = 1) we explored associations between Autism traits and demographic factors. We found no statistically significant differences between participants with autism traits and those without in relation to the following demographic factors: age (*x* ^**2**^ = 1.53**;** *p* = 0.22), sex assigned at birth (*x* ^**2**^ = 0.62; *p* = 0.43), self-identification (*x* ^**2**^ = 5.22; *p* = 0.16), respondent education (*x* ^**2**^ = 5.02; *p* = 0.08), respondent occupation (*x* ^**2**^ = 2.67; *p* = 0.45), family occupation (*x* ^**2**^ = 2.80; *p* = 0.42), sexual orientation (*x* ^**2**^ = 3.88; *p* = 0.28), cross-sex hormone use (*x* ^**2**^ = 0.73; *p* = 0.39), gender affirming surgical procedures (*x* ^**2**^ = 0.76; *p* = 0.39), wish to access cross-sex hormones (*x* ^**2**^ = 0.18; *p* = 0.68), and wish to access gender affirming surgery (*x* ^**2**^ = 1.90; *p* = 0.17).

**Table 2.** Sociodemographic, sexual orientation, and clinical intervention characteristics associated with autism traits.

		All *N* (%)	Autism traits *N* (%)		
			No	Yes	*X* ^2^ (*df* = 1)
			101 (82.8%)	21 (17.2)	Significance
Current age (total = 123)	Adolescents < 18	38 (30.9)	33 (32.7)	4 (19.1)	*X* ^**2**^ = 1.53
	Adults > 18	85 (69.1)	68 (67.3)	17 (81.0)	*p* = 0.22
Sex at birth (total = 123)	Male	57 (46.3)	48 (47.5)	8 (38.1)	*X* ^**2**^ = 0.62
	Female	66 (53.7)	53 (52.5)	13 (61.9)	*p* = 0.43
Self-identified Gender (total = 123)	Male	42 (34.2)	32 (31.7)	10 (47.6)	*X* ^**2**^ = 5.22
	Female	33 (26.8)	27 (26.7)	6 (28.6)	*p* = 0.16
	Transmale/transfemale	42 (34.2)	38 (37.6)	3 (14.3)	
	Non-binary	6 (4.9)	4 (3.97)	2 (9.5)	
Respondent Education (total = 118)	Degree or higher	6 (5.1)	3 (3.1)	3 (15.0)	*X* ^**2**^ = 5.02
	Intermediate	83 (70.3)	71 (72.5)	12 (60.0)	*p* = 0.08
	No qualifications	29 (24.6)	24 (24.5)	5 (25.0)	
Respondent occupational social class (total = 118)	Professional	33 (28.0)	13 (13.3)	5 (25.0)	*X* ^**2**^ = 2.67
	Intermediate	19 (16.1)	5 (5.1)	0 (0.0)	*p* = 0.45
	(Semi-)routine	35 (29.7)	24 (24.5)	4 (20.0)	
	Other	31 (26.3)	56 (57.1)	11 (55.0)	
Occupational social class of family (total = 121)	Professional	53 (43.8)	42 (42.0)	11 (52.4)	*X* ^**2**^ = 2.80
	Intermediate	23 (19.0)	21 (21.0)	2 (9.5)	*p* = 0.42
	(Semi-)routine	39 (32.2)	33 (33.0)	6 (28.6)	
	Other	6 (5.0)	4 (4.0)	2 (9.5)	
Sexual orientation (total = 121)	Monosexual	48 (39.7)	42 (42.4)	6 (28.6)	*X* ^**2**^ = 3.88
	Plurisexual	58 (47.9)	45 (45.5)	13 (61.9)	*p* = 0.28
	Asexual	7 (5.8)	5 (5.1)	2 (9.5)	
	Other/unsure	8 (6.6)	7 (7.1)	0 (0.0)	
Cross-sex hormones (total = 122)	No	74 (60.7)	63 (62.4)	11 (52.4)	*X* ^**2**^ = 0.73
	Yes	48 (39.3)	38 (37.6)	10 (47.6)	*p* = 0.39
Gender affirming surgery (total = 121)	No	95 (78.5)	80 (80.0)	15 (71.4)	*X* ^**2**^ = 0.76
	Yes	26 (21.5)	20 (20.0)	6 (28.6)	*p* = 0.39
Desire to access cross-sex hormones (total = 122)	Yes	118 (96.7)	98 (97.0)	20 (95.2)	*X* ^**2**^ = 0.18
	No	4 (3.3)	3 (3.0)	1 (4.8)	*p* = 0.68
Desire to have gender affirming surgery (total = 122)	Yes	117 (95.9%)	98 (97.0%)	19 (90.5%)	*X* ^**2**^ = 1.90
	No	5 (4.1%)	3 (3.0%)	2 (9.5%)	*p* = 0.17

## Discussion

This study explored socio-demographic, sexual orientation and clinical intervention characteristics of participants. As treatment status was not controlled for, participants were at various stages in relation to accessing gender affirming interventions. AFAB individuals were younger compared to AMAB individuals, which was reflected in younger mean ages of self-identified males, transmen, and non-binary individuals. Comparing respondent family occupational social class and respondent own occupational social class highlighted that a lower proportion of respondents were in the professional, intermediate, and (semi)-routine category, and a higher proportion of respondents in the other category compared to family occupational social class.

Sexual orientation was not linked to age, with participants of all ages identifying across a range of sexual orientations.

While the wish to access cross-sex hormones and gender affirming surgical interventions was independent of age; cross-sex hormone use and access to gender affirming surgeries was linked to age. Adolescents are usually prescribed cross-sex hormones after a period of time on hormone blockers, while adults can access cross-sex hormones in conjunction with hormone blockers or on their own without time on hormone blockers only. This may partly explain why adolescents in this study had not accessed cross-sex hormones. Gender affirming surgical interventions are currently available to adults only, often after a period of time on cross-sex hormones, which explains the older mean ages of participants who had accessed gender affirming surgical procedures.

This study used a range of diagnostic tools to examine autism traits in a population of adolescent and adult individuals seeking gender-affirming treatments. Combining three different autism trait screening tools allowed a more stringent assessment than using only one. We found higher rates of autism traits than previous studies in Belgium and the UK which reported prevalence of 4.84–5.5%, respectively [30,29] based on the AQ, but lower rates than Nobili et al. [32] who reported a prevalence of 36.3% based on the AQ-short. The considerable differences in autism prevalence may be explained by variations in access criteria for specialist gender services. As adult studies used self-report rather than parental report measures or diagnostic assessments, individuals may have been reluctant to answer questions about autism traits truthfully for fear of this impacting on their ability to access gender affirming interventions. Compared to other studies with adolescents Di Ceglie et al. [19], we found no differences in mean differences between AMAB and AFAB individuals in relation to empathy levels. Compared to other studies with adolescents Kaltiala-Heino et al. [25] based on clinical chart data, we found lower levels of Autism traits using screening tools.

This study highlights a population seeking gender affirming treatments who have significant social and communication difficulties. There was no significant difference between those with and without autism traits in relation wanting access to gender-affirming hormonal treatments. Only a small minority (*N* = 3) with Autism traits did not wish to have surgical interventions. Therefore, we must assume that individuals with autism traits present to services for access to interventions. Based on findings in our study, it is likely that a significant number of individuals seeking gender affirming interventions from services in the future will present with autism traits. Clinicians involved in service provision require the skills to recognize and screen for autism traits. The use of a combination of screening tools could identify autism traits in a quick and cost-effective way. Future commissioning of services requires robust pathways in which individuals can access autism specific services alongside their gender affirming treatments. Future longitudinal research studies could examine the outcomes and experiences of individuals with autism traits over longer periods of time.

## Limitations

There are several limitations in this paper. A small sample size, limitations related to recruitment method and a sample containing both adolescents and adults may hinder generalizability of findings. Treatment status may have also impacted on findings. Without a comparison group we cannot be certain whether high rates of autism traits are evident only in those seeking gender affirming treatments or whether this is a feature in the general population.

## Data Availability

Due to the sensitive nature of this study, ethical agreements do not allow public sharing of the research data. We are happy to facilitate any queries or questions related to the research as per ethical agreements.
